# Water‐mediated interactions destabilize proteins

**DOI:** 10.1002/pro.4168

**Published:** 2021-08-20

**Authors:** Tomonari Sumi, Hiroshi Imamura

**Affiliations:** ^1^ Research Institute for Interdisciplinary Science Okayama University Kita‐ku Japan; ^2^ Department of Chemistry, Faculty of Science Okayama University Kita‐ku Japan; ^3^ Department of Applied Chemistry, College of Life Sciences Ritsumeikan University Kusatsu Japan

**Keywords:** hydrophobic interactions, intramolecular and intermolecular dispersion forces, protein folding stability, solvation‐free energy, water‐mediated interactions

## Abstract

Proteins are folded to avoid exposure of the nonpolar groups to water because water‐mediated interactions between nonpolar groups are a promising factor in the thermodynamic stabilities of proteins—which is a well‐accepted view as one of the unique effects of hydrophobic interactions. This article poses a critical question for this classical view by conducting an accurate solvation free‐energy calculation for a thermodynamic cycle of a protein folding using a liquid‐state density functional theory. Here, the solvation‐free energy for a leucine zipper formation was examined in the coiled‐coil protein GCN4‐p1, a typical model for hydrophobic interactions, which demonstrated that water‐mediated interactions were unfavorable for the association of nonpolar groups in the native state, while the dispersion forces between them were, instead, responsible for the association. Furthermore, the present analysis well predicted the isolated helical state stabilized by pressure, which was previously observed in an experiment. We reviewed the problems in the classical concept and semiempirical presumption that the energetic cost of the hydration of nonpolar groups is a driving force of folding.

## 1 INTRODUCTION

**FIGURE 1:**
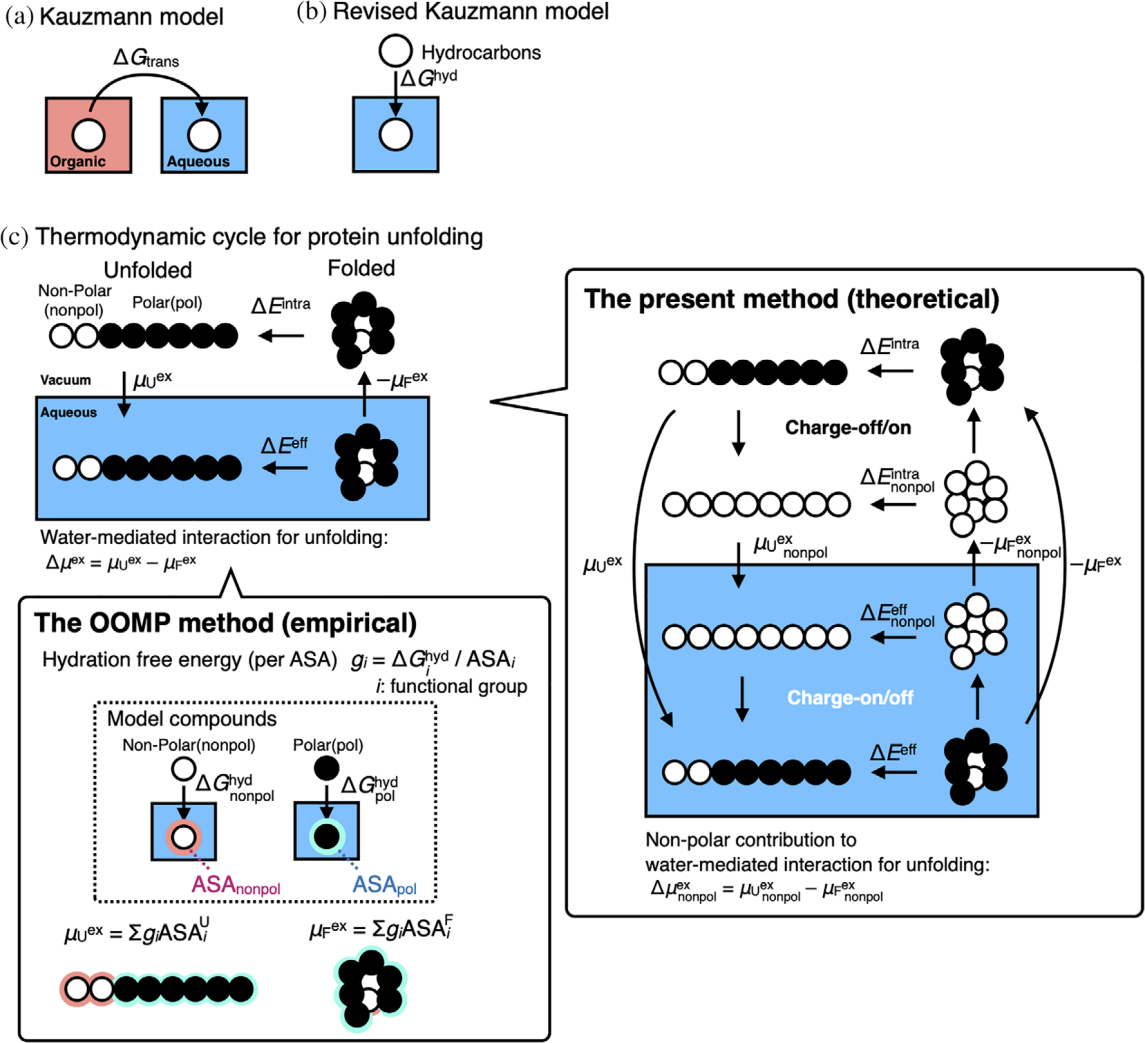
Thermodynamic description of protein unfolding. (a) In the 1959 Kauzmann model,^8^ protein unfolding, which is accompanied by water‐exposure of the nonpolar groups buried inside a folded protein, is regarded as transfer of nonpolar hydrocarbons from an organic solvent into water with the transfer Gibbs free energy (Δ*G*_trans_). (b) In the revised Kauzmann model,^15^, ^16^ gas‐to‐water transfer of the nonpolar hydrocarbons with the hydration Gibbs free energy (Δ*G* ^hyd^) resembles the protein unfolding. (c) Thermodynamic cycle for protein unfolding, where protein unfolding energetics in water and in vacuum are Δ*E* ^eff^ and Δ*E* ^intra^, respectively, and the hydration free energies of the folded and unfolded conformations are *μ*_F_^ex^ and *μ*_U_^ex^, respectively. Δ*μ* ^ex^ (= *μ*_U_^ex^ –*μ*_F_^ex^) indicates the water‐mediated interaction for unfolding, which is calculated empirically by the OOMP method (see the details in the text, the Appendix, and the literature^2^, ^3^, ^17^, ^18^, ^19^, ^20^, ^21^) or theoretically by the present method. In the present method, the nonpolar part contributions ($\mu_{F,\text{nonpol}}^{\mathrm{ex}}$ and $\mu_{U,\text{nonpol}}^{\mathrm{ex}}$) to the hydration free energies are given by off the charges of the protein molecule. ${∆\mu }_{\text{nonpol}}^{\mathrm{ex}}\;(=\mu_{U,\text{nonpol}}^{\mathrm{ex}}{-\mu }_{F,\text{nonpol}}^{\mathrm{ex}}$) is the nonpolar part contribution to the water‐mediated interaction

Quantitative understanding of the energetics of protein folding or of the dominant factor behind the thermodynamic stability of proteins remains a challenging issue in biochemistry, biophysics, and molecular biology.^1^, ^2^, ^3^, ^4^, ^5^, ^6^ Amino acid residues, which form a polypeptide chain in proteins, are classified as hydrophobic and hydrophilic, and the former has lower solubility than the latter in water, but better solubility in organic solvents than in water.^7^ In 1959, Kauzmann proposes in his seminal review^8^ that dehydration of nonpolar groups, followed by their association, is energetically favorable and thereby this “hydrophobic bond,” referred to as a “hydrophobic interaction” later,^9^ should be the dominant factor in the thermodynamic stability of protein conformations. The Kauzmann hydrophobic interaction hypothesis has attracted many scientists and has been widely accepted for ~60 years.^1^, ^5^, ^6^, ^7^, ^10^, ^11^, ^12^ This hypothesis is based on the free energy for the transfer of nonpolar hydrocarbons from water into a nonaqueous solution or into its own neat liquid (Figure 1a). The free energy for the transfer is a negative value and is supposed to mimic the change upon burying nonpolar groups exposed in water into a folded protein interior.^3^, ^4^ While a “hydrophobic interaction” has been loosely defined, it actually includes two interactions: van der Waals interactions between nonpolar groups (dispersion forces), and an effective, water‐mediated interaction (hydration effect).^13^ Nonetheless, the latter water‐mediated interaction has been preferentially appreciated as a favorable interaction for protein folding.^11^, ^12^ In fact, a “hydrophobic interaction” is generally introduced in the text books of molecular biology as follows: water forces hydrophobic groups together because the apparent attraction between them is actually caused by a repulsion from water.^11^, ^12^ Often, a “hydrophobic interaction” has been described as an entropically favorable interaction, the origin of which could be a special water structure around a nonpolar solute, which has also been argued as an issue.^14^

In the Kauzmann model of protein folding, the protein interior is treated as an organic liquid; thus, it has often been claimed that such a protein model is coarse‐grained, oversimplified, and thus, unrealistic.^4^ The exact correlation between the thermodynamics of protein folding and the energetics of the hydrocarbon transfer also remains controversial.^22^, ^23^ In 2013 and 2014, Baldwin revised the Kauzmann model (denoted here as the revised Kauzmann model); he divided the transfer free energy into two contributions in the liquid‐to‐gas and gas‐to‐water processes (Figure 1b). The article discusses the positive free energy for the latter gas‐to‐water transfer of nonpolar model compounds to analyze protein folding energetics.^15^, ^16^ The revised Kauzmann model presumes that unfavorable hydration (gas‐to‐water) of nonpolar groups acts as a stabilizing factor of the folded structure of proteins, in which the van der Waals interactions between nonpolar groups are dismissed (see Figure 1b). Although this model is thought‐provoking and intuitively appealing, it also looks oversimplified. The discussion according to the analogy between protein unfolding and the gas‐to‐water transfer of nonpolar groups does not appreciate a hydration of the folded protein (*μ*_F_^ex^ in Figure 1c), which requires unfavorable cavity formation.

Apart from these classical coarse‐grained models, the introduction of a thermodynamic cycle, as shown in Figure 1c, enables the decomposition of the free energy for protein unfolding into the free energy in a vacuum and the hydration‐free energy (also denoted as solvation‐free energy in this article). Protein unfolding energetics in water (Δ*E* ^eff^) is equivalent to the sum of the energetics for (i) dehydration of the folded protein (–*μ*_F_^ex^), (ii) unfolding in the gas phase (Δ*E* ^intra^), and (iii) hydration of the unfolded protein (*μ*_U_^ex^). Thus, this cycle determines how the water‐mediated interaction acts, given as a difference in the hydration‐free energy between the folded and unfolded conformations (Δ*μ* ^ex^ = *μ*_U_^ex^ –*μ*_F_^ex^). Because reliable *μ*_F_^ex^ and *μ*_U_^ex^ had been beyond the reach of computations, the earliest method by Ooi and Oobatake^2^, ^17^, ^18^ and by Makhatadze and Privalov^3^, ^20^, ^21^ (the OOMP method) empirically presumed that the hydration‐free energies (*μ*_F_^ex^ and *μ*_U_^ex^) were proportional to the accessible surface area (ASA) of the protein (see Figure 1c, the Appendix, and related literature^2^, ^3^, ^17^, ^18^, ^19^, ^20^, ^21^). Interestingly, the authors found that the water‐mediated interaction destabilizes the folded conformations of proteins.^2^, ^3^, ^18^ Makhatadze and Privalov^3^ elaborated on the group contributions to the hydration Gibbs energy for protein unfolding per ASA as follows: polar surface, −1,000 ± 50 J mol^−1^ Å^−2^; aliphatic surface, 50 J mol^−1^ Å^−2^; aromatic surface, −53 J mol^−1^ Å^−2^ at 25°C. Intramolecular interactions, such as van der Waals interactions, between nonpolar groups and the hydrogen bonds—direct interaction—stabilize the folded conformations of proteins, as an alternative to unfavorable water‐mediated interactions (hydration effects)—indirect interactions. Accordingly, the researchers who follow these studies^2^, ^3^, ^18^ have appreciated rather minor contributions of nonpolar (aliphatic and aromatic) groups to the hydration Gibbs energy for protein unfolding. The classical Kauzmann hypothesis, that is, that the energetic cost of the hydration of nonpolar groups is a dominant factor for folding, has been weakened, but still appeals to us to accept that the exposure of aliphatic nonpolar groups is avoided to stabilize the folded conformation. This is because the OOMP method determines water‐mediated interactions between nonpolar groups to be favorable.

On the other hand, emerging evidence indicates that the water‐mediated interaction of nonpolar groups could be favorable and unfavorable, depending on their sizes and shapes.^24^, ^25^ For example, the water‐mediated interaction at 298 K is attractive between neopentane, while it is repulsive between bicyclooctane, adamantane, and fullerene.^24^ This reveals the limitation of the OOMP method, that is, the ASA‐based extrapolation of the hydration Gibbs free energy using data of simple model compounds such as alkanes. The OOMP method regards buried groups without ASA as noncontributors to the hydration Gibbs energy of protein, so that the positive cavity formation energy for the buried groups is dismissed. These biases toward the unreasonable stabilization of the folded conformation with a lesser ASA of buried aliphatic nonpolar groups (see details in the Appendix).

It needs, thus, a more rigorous and theoretical method to address whether the water‐mediated interaction of nonpolar groups stabilize proteins. Recently, a new computational method using the reference‐modified density functional theory (RMDFT) has been developed for accurate and efficient hydration free‐energy calculations, which has been applied to ab‐initio analysis of the thermodynamic stability of a designed small 10‐amino‐acid‐residue protein, chignolin, according to the thermodynamic cycle.^26^ As depicted in Figure 1c, this method can decompose the nonpolar part contributions ($\mu_{F,\text{nonpol}}^{\mathrm{ex}}$ and $\mu_{U,\text{nonpol}}^{\mathrm{ex}}$) to the hydration free energies (*μ*_F_^ex^ and *μ*_U_^ex^) by off the charges of the protein molecule, which provides the nonpolar part contribution (${∆\mu }_{\text{nonpol}}^{\mathrm{ex}}=\mu_{U,\text{nonpol}}^{\mathrm{ex}}{-\mu }_{F,\text{nonpol}}^{\mathrm{ex}}$) to the water‐mediated interaction (Δ*μ* ^ex^ = *μ*_U_^ex^ –*μ*_F_^ex^). The study on chignolin demonstrated that the water‐mediated interactions (the total and the nonpolar part contributions) destabilized the folded state of chignolin while the intramolecular direct interactions (van der Waals interactions between nonpolar groups and hydrogen bonds) predominantly stabilized the folded state. The latter slightly overcame the former, resulting in the stabilization of the folded state, which is termed as a direct interaction mechanism. This mechanism is clearly in contrast to Kauzmann's hydrophobic interaction hypothesis and also distinct from the picture by the OOMP method. The small size of chignolin among proteins, however, might hinder the application of the conclusion to general proteins; the folding energetics of chignolin without fully buried nonpolar residues might be different from that of larger and more complicated proteins with hydrophobic interior cores.

In the present study, a theoretical analysis of the hydration‐free energy change upon leucine zipper formation in GCN4‐p1 has been conducted using the RMDFT.^27^, ^28^, ^29^, ^30^ GCN4‐p1 forms a parallel, two‐stranded coiled coil of *α*‐helices^31^, ^32^ packed in the “knobs‐into‐holes” manner proposed by Crick in 1953.^33^ GCN4‐p1 with nonpolar interfaces consisting of a leucine repeat^31^, ^32^ is one of the most typical model proteins^34^ whose folded states are believed to be stabilized by the water‐mediated interactions of “hydrophobic interaction.^35^” However, the present study on GCN4‐p1 demonstrates that the water‐mediated interactions of the total and nonpolar part contribution destabilize the protein. This work will boost change in the current paradigm that proteins are stabilized by avoiding exposure of the nonpolar groups to water.

## 2 METHODS

**FIGURE 2:**
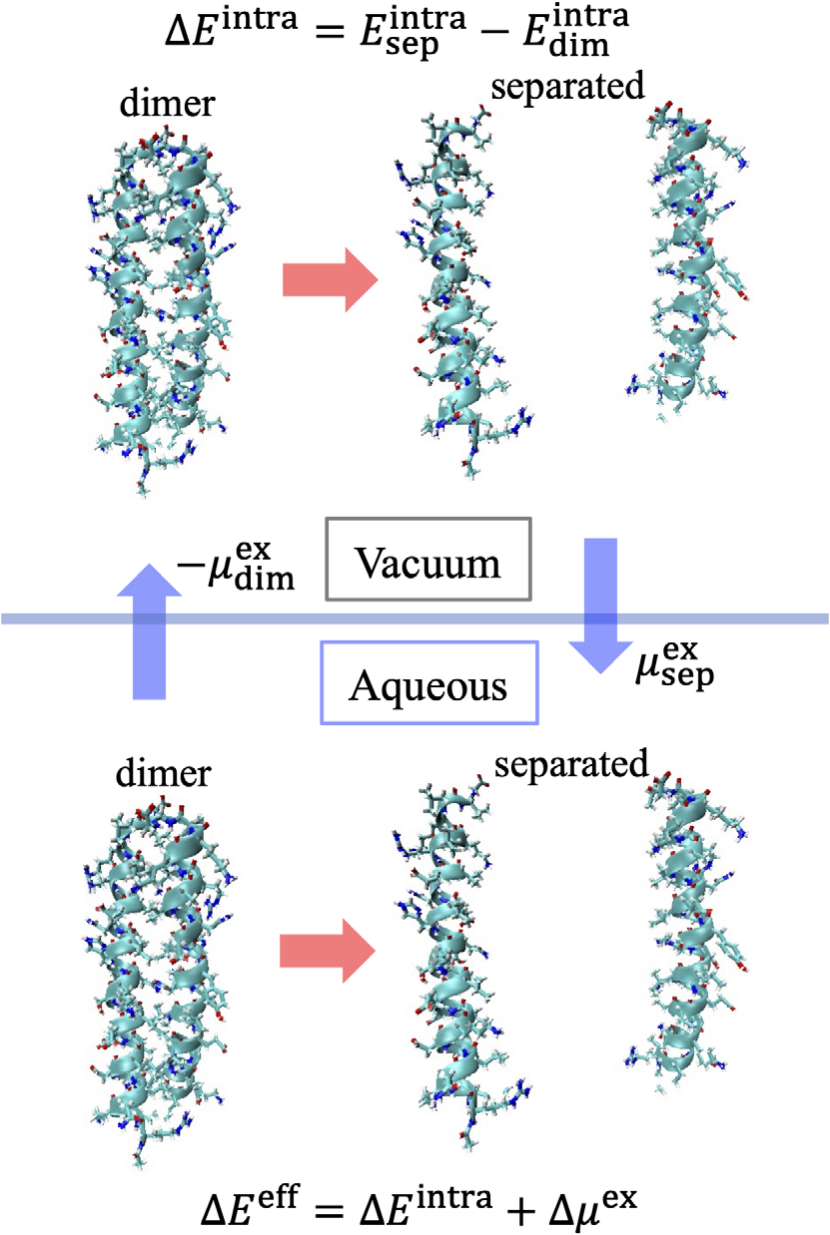
Thermodynamic cycle for investigating energetics of GCN4‐p1 leucine zipper interactions. The effective energy $E^{\mathrm{eff}}$, defined as a sum of the intramolecular interaction energy $E^{\text{intra}}$ and the solvation‐free energy (hydration free energy) $\mu^{\mathrm{ex}}$, is calculated for the dimer state and the separated state. Using these values, changes in these energies upon dissociation of the two‐stranded coiled coil helices, $∆E^{\mathrm{eff}}$, $∆E^{\text{intra}}$, and $∆\mu^{\mathrm{ex}}$, are determined

The thermodynamic cycle shown in Figure 2 has been applied to investigate the energetics of the GCN4‐p1 leucine zipper interaction. To generate conformations for the parallel two‐stranded coiled coil helices, an all‐atom‐model molecular dynamics (MD) simulation of GCN4‐p1 was performed in water for 1 μs. Using the generated MD conformations, the ensemble average of the intramolecular energy $E_{\dim}^{\text{intra}}={\left\langle E_{i}^{\text{intra}}\right\rangle }_{\dim}$, solvation‐free energy $\mu_{\dim}^{\mathrm{ex}}={\left\langle \mu_{i}^{\mathrm{ex}}\right\rangle }_{\dim}$, and effective energy $E_{\dim}^{\mathrm{eff}}={\left\langle E_{i}^{\mathrm{eff}}\right\rangle }_{\dim}$, where the effective energy is defined as $E_{i}^{\mathrm{eff}}=E_{i}^{\text{intra}}+\mu_{i}^{\mathrm{ex}}$, and ${\left\langle \;\right\rangle }_{\dim}$ is the ensemble average provided by the dimer conformations, has been calculated. Hereafter, this is referred to as the “dimer” state. To calculate the change in the effective energy upon dissociation of the leucine zipper interaction, helical monomer conformations were prepared by removing one of the coiled coil helices. Such a generation scheme has been employed because an isolated helix monomer of GCN4‐p1 is unstable at room temperature, atmospheric pressure, and transforms into a coil.^36^ In the same way, using the generated monomeric helical conformations, $E_{\mathrm{sep}}^{\text{intra}}=2{\left\langle E_{i}^{\text{intra}}\right\rangle }_{\text{helix}}$, $\mu_{\mathrm{sep}}^{\mathrm{ex}}=2{\left\langle \mu_{i}^{\mathrm{ex}}\right\rangle }_{\text{helix}}$, and $E_{\mathrm{sep}}^{\mathrm{eff}}=2{\left\langle E_{i}^{\mathrm{eff}}\right\rangle }_{\text{helix}}$, where ${\left\langle \;\right\rangle }_{\text{helix}}$ is the ensemble average using monomeric helical conformations, have been calculated. Hereafter, this is referred to as the “separated” state. From these thermodynamic quantities, the change in the effective energy upon unbinding of the coiled coil helices, $∆E^{\mathrm{eff}}=E_{\mathrm{sep}}^{\mathrm{eff}}-E_{\dim}^{\mathrm{eff}}$, and its decomposition into the two terms, $∆E^{\text{intra}}=E_{\mathrm{sep}}^{\text{intra}}-E_{\dim}^{\text{intra}}$ and $∆\mu^{\mathrm{ex}}=\mu_{\mathrm{sep}}^{\mathrm{ex}}-\mu_{\dim}^{\mathrm{ex}}$ may be calculated.

Here, it is useful to note that the effective energy $E_{i}^{\mathrm{eff}}$ appears in the partition function for a protein immersed in water, in which $E_{i}^{\mathrm{eff}}$ gives the Boltzmann factor of this system. Therefore, $∆E^{\mathrm{eff}}$ and its components, $∆E^{\text{intra}}$ and $∆\mu^{\mathrm{ex}}$, provide decisive insights into the thermodynamic stability of proteins, as argued in a previous study.^37^ In fact, the recent study for the model protein chignolin^26^ demonstrates that such an effective energy analysis using $∆E_{i}^{\mathrm{eff}}$, $∆E_{i}^{\text{intra}}$, and $∆\mu_{i}^{\mathrm{ex}}$ ^30^ derives the correct conclusion to the energetics of the thermodynamic stability of chignolin.

In addition, to investigate the energetics of folding stability from the unfolded coil state to the dimer state, an all‐atom‐model MD simulation was performed of the monomer coil in water for 1 μs. In the same way, using the generated monomer coil conformations, the change in the effective energy upon unfolding of the coiled coil helices into two coils, $∆E^{\mathrm{eff}}=2{\left\langle E_{i}^{\mathrm{eff}}\right\rangle }_{\text{coil}}-E_{\dim}^{\mathrm{eff}}$, and its decomposition into two terms, that is, the enthalpy term in a vacuum, $∆E^{\text{intra}}=2{\left\langle E_{i}^{\text{intra}}\right\rangle }_{\text{coil}}-E_{\dim}^{\text{intra}}$, and the water‐mediated interaction, $∆\mu^{\mathrm{ex}}=2{\left\langle \mu_{i}^{\mathrm{ex}}\right\rangle }_{\text{coil}}-\mu_{\dim}^{\mathrm{ex}}$, where ${\left\langle \;\right\rangle }_{\text{coil}}$ is the ensemble average using the generated monomeric coil conformations, have been calculated.

## 3 RESULTS AND DISCUSSION

**FIGURE 3:**
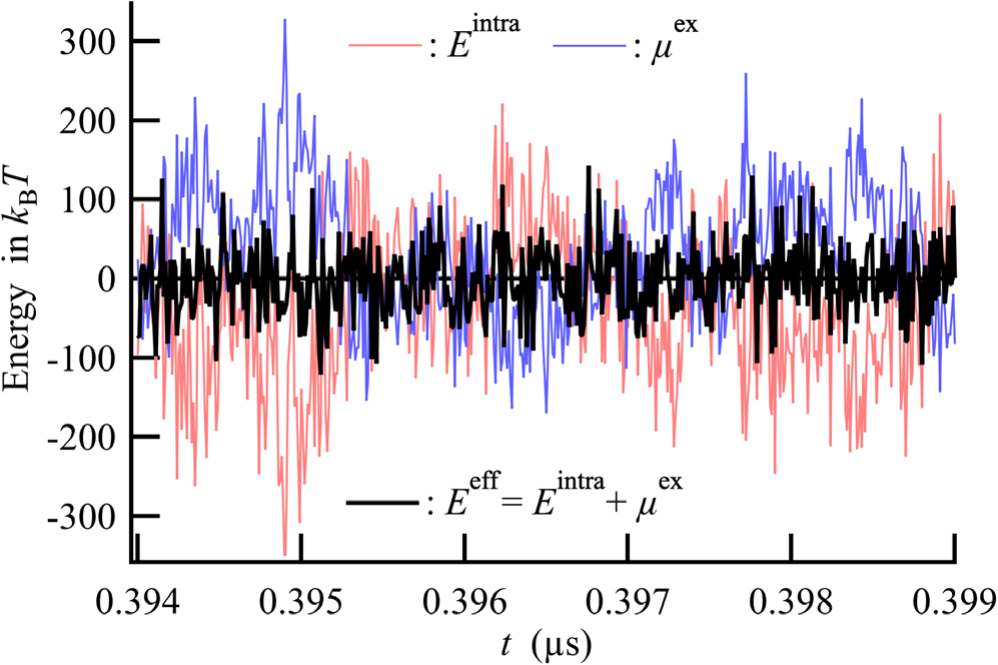
Thermal fluctuation of $E_{i}^{\text{intra}}$, $\mu_{i}^{\mathrm{ex}}$, and $E_{i}^{\mathrm{eff}}$ for two‐stranded GCN4‐p1 coiled coil helices as a function of time at room temperature and atmospheric pressure. Time course of $E_{i}^{\text{intra}}$, $\mu_{i}^{\mathrm{ex}}$, and $E_{i}^{\mathrm{eff}}$ is shifted by minus the mean value. The standard deviations of $E_{i}^{\text{intra}}$, $\mu_{i}^{\mathrm{ex}}$, and $E_{i}^{\mathrm{eff}}$ are 141, 116, and 46 in $k_{B}T$, respectively

The native structure of proteins in aqueous solution largely fluctuates thermally; thus, the fluctuations of $E_{i}^{\text{intra}}$ and $\mu_{i}^{\mathrm{ex}}$ are much larger than $k_{B}T$. In fact, the time course of $E_{i}^{\text{intra}}$ and $\mu_{i}^{\mathrm{ex}}$ obtained for the two‐stranded GCN4‐p1 coiled coil helices in water varied to a large extent, up to $\sim \pm 200k_{B}T$ from the mean value (Figure 3). $E_{i}^{\text{intra}}$ and $\mu_{i}^{\mathrm{ex}}$ were obviously anticorrelated to each other, indicating that these contributions largely compensated each other in $E_{i}^{\mathrm{eff}}$. This observation showed that intramolecular and water‐mediated interactions acted as competitive factors upon forming an energy basin for the native state of the protein. The standard deviations of $E_{i}^{\text{intra}}$ and $\mu_{i}^{\mathrm{ex}}$ were 141 and 116 in $k_{B}T$, respectively, while that of $E_{i}^{\mathrm{eff}}$ was obtained as 46 in $k_{B}T$. Therefore, an ensemble average over a long time was required to quantitatively discuss the energetics of the leucine zipper interaction for GCN4‐p1. In this study, molecular dynamics (MD) simulations of both the two‐stranded GCN4‐p1 coiled coil helices and its isolated monomeric coil were performed, in water for 1 μs, and the ensemble average of these thermodynamic quantities was calculated using 10^5^ conformations generated by the 1μs‐MD simulations.

### 3.1 Energetics of the leucine zipper interaction

**FIGURE 4:**
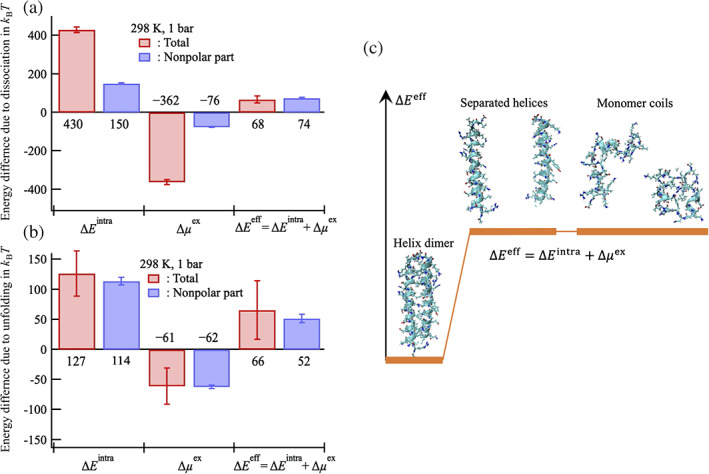
Change in the effective energy upon (a) dissociation of the GCN4‐p1 coiled coil helices, $∆E^{\mathrm{eff}}=E_{\mathrm{sep}}^{\mathrm{eff}}-E_{\dim}^{\mathrm{eff}}$, and (b) upon unfolding of GCN4‐p1 into two isolated coils, $∆E^{\mathrm{eff}}=2{\left\langle E_{i}^{\mathrm{eff}}\right\rangle }_{\text{coil}}-E_{\dim}^{\mathrm{eff}}$, at 298 K, 1 bar, and these decompositions into the intramolecular interaction part $∆E^{\text{intra}}$ and the water‐mediated part $∆\mu^{\mathrm{ex}}$. Furthermore, these quantities are decomposed into nonpolar and polar parts. The nonpolar part of $∆E^{\text{intra}}$, $∆\mu^{\mathrm{ex}}$, and $∆E^{\mathrm{eff}}$, namely $∆E_{\text{nonpol}}^{\text{intra}}$, $∆\mu_{\text{nonpol}}^{\mathrm{ex}}$, and $∆E_{\text{nonpol}}^{\mathrm{eff}}$, which are calculated by eliminating all electric charges on GCN4‐p1, are also shown. The present nonpolar part contributions include the contribution by all polar/nonpolar groups in the protein and are different from what the earlier studies^2^, ^3^, ^17^, ^18^, ^19^, ^20^, ^21^ stated (detailed in the text). In (a) and (b), the number shown below/above each bar indicates the value of these thermodynamic quantities (also see Tables S1 and S2). Standard errors (the definition is presented in the main text) are shown as the error bars in (a) and (b). As depicted schematically in (c), both the separated and coil state are more unstable than the dimer state, whereas $∆E^{\mathrm{eff}}$ for these states are nearly comparable. Previous literature also indicates that the isolated two‐stranded coiled‐coil helices are stable^44^

The energetics upon unbinding of the leucine zipper interaction at 298 K, 1 bar is summarized in Figure 4a as a category plot (also see Table S1). All standard errors shown in this study were evaluated by decomposing the 10^5^‐conformation average into five equal block averages. The standard errors indicated as error bars were smaller than the physical quantity values resulting from the ensemble average by a total of 10^5^ conformations. The intramolecular interaction energy increased by $430k_{B}T$ and the solvation‐free energy decreased by $362k_{B}T$ when the coiled coil helical dimer was separated into two helical monomers. Consequently, the effective energy increased by $68k_{B}T$. Furthermore, by eliminating all electric charges on GCN4‐p1, the nonpolar part contributions to these quantities were calculated, namely, $∆E_{\text{nonpol}}^{\text{intra}}$, $∆\mu_{\text{nonpol}}^{\mathrm{ex}}$, and $∆E_{\text{nonpol}}^{\mathrm{eff}}$ (see the schematic explanation in Figure 1c). Figure 4a shows that the absolute values of $∆E_{\text{nonpol}}^{\text{intra}}$ and $∆\mu_{\text{nonpol}}^{\mathrm{ex}}$ were less than one‐third of the absolute values of $∆E^{\text{intra}}$ and $∆\mu^{\mathrm{ex}}$, respectively, while these smaller nonpolar parts yielded values $∆E_{\text{nonpol}}^{\mathrm{eff}}$ that were comparable with $∆E^{\mathrm{eff}}$. The dispersion interaction between the nonpolar helices increased by $150k_{B}T$ and the nonpolar part of the water‐mediated interaction decreased by $76k_{B}T$. As a result, the nonpolar part of $∆E^{\mathrm{eff}}$ increased by $74k_{B}T$. Hydrophobic interactions consist of water‐mediated interactions and van der Waals interactions between nonpolar groups; the latter is the dispersion force included in the van der Waals interaction,^38^ which works for nonpolar molecules. A comparable $∆E_{\text{nonpol}}^{\mathrm{eff}}$ value to the total $∆E^{\mathrm{eff}}$ value reflects the importance of the contribution of the nonpolar part $∆E_{\text{nonpol}}^{\mathrm{eff}}$, that is, van der Waals interactions between nonpolar groups in the leucine zipper interaction. The negative value of $∆\mu_{\text{nonpol}}^{\mathrm{ex}}$, as well as $∆\mu^{\mathrm{ex}}$, indicates that the water‐mediated interaction between the nonpolar parts in the helices is unfavorable, that is, the so‐called hydrophobic hydration—hydration of nonpolar moieties effect stabilized the separated state. We note that the present method does not decompose the nonpolar *group contributions* but extracts nonpolar *part contributions*. Former “*group contribution*” analyses, such as the OOMP method,^2^, ^3^, ^17^, ^18^, ^19^, ^20^, ^21^ assume that the context dependence (e.g., the location of the group in the molecule) is negligible, and the thermodynamic quantities of the whole molecule are given by summing the quantities of the constituting groups. The latter analysis extracts the nonpolar part contributions by turning off the electric charges of all polar/nonpolar groups in the protein (Figure 1c); the effects of charge‐off on the solvation‐free energy of amino acid side chain analogues are demonstrated in Figure S1 in the SI. Accordingly, the comparison of $∆\mu_{\text{nonpol}}^{\mathrm{ex}}$ with the relevant hydration effects in earlier studies^2^, ^3^, ^17^, ^18^, ^19^, ^20^, ^21^ is not straightforward.

A similar unfavorable water‐mediated interaction has been observed for the large hydrocarbon molecules bicyclooctane and adamantine,^24^ fullerene C_60_,^24^, ^39^ carbon nanotubes, and graphene sheets.^40^ Interestingly, the osmotic second virial coefficient of the smallest hydrocarbon, methane, indicates the unfavorable water‐mediated interaction between methane molecules at room temperatures.^41^ It was noted that the values of the nonpolar part of the solvation‐free energy for the dimer and the separated state of the helices were positive in the same manner as the gas–liquid transfer free energy of hydrocarbons (see Table S1), while the water‐mediated interaction $∆\mu_{\text{nonpol}}^{\mathrm{ex}}$ did not necessarily stabilize the dimer state. This observation is a seemingly counter‐intuitive result and contradicts the underlying assumption in the argument of hydrophobic factors on protein stability by Baldwin.^15^, ^16^, ^42^, ^43^ The results presented here demonstrated that the dominant factor in the leucine zipper interaction and the physical origin was not the water‐mediated interaction between nonpolar groups, but the van der Waals interactions between them.

### 3.2 Energetics of GCN4‐p1 folding

Energetics upon the transition from the folded dimeric GCN4‐p1 to two monomeric coils at 298 K and 1 bar are summarized in Figure 4b as a category plot (also see Table S2). The change in these thermodynamic quantities upon unfolding was similar to that obtained for the leucine zipper interaction shown in Figure 4a. However, in contrast to the energetics for the dissociation, the predominant component for the change in $∆E^{\text{intra}}$ and $∆\mu^{\mathrm{ex}}$ upon unfolding was the nonpolar part; thus, the polar part provided a minor contribution to $∆E^{\text{intra}}$ and $∆\mu^{\mathrm{ex}}$. Interestingly, as depicted schematically in Figure 4c, $∆E^{\mathrm{eff}}$ for the coil state was almost comparable to $∆E^{\mathrm{eff}}$ for the separated state, indicating that the separated state does not necessarily work as an intermediate state for leucine zipper formation. The nonpolar part of $∆\mu^{\mathrm{ex}}$, as well as $∆\mu^{\mathrm{ex}}$ itself, stabilized the unfolded coil state (Figure 4b), thus indicating an evident contradiction to the presumption of the favorable water‐mediated interaction for folding as seen in the Kauzmann hypothesis. As previously claimed using chignolin as the model protein,^26^, ^30^ the intramolecular direct interaction mechanism on the thermodynamic stability of the protein was demonstrated again: the native structure depended on the competition between intramolecular direct and indirect (water‐mediated) interactions, and the former slightly overcame the latter, thereby resulting in the thermodynamic stability of the folded conformations for the native state.

### 3.3 Effect of pressure on GCN4‐p1 thermodynamic stability

**FIGURE 5:**
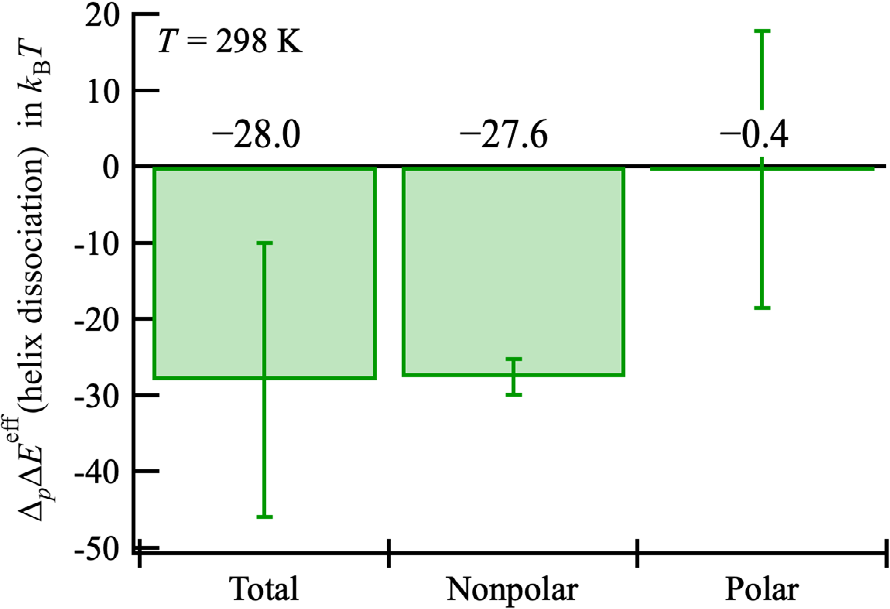
Effect of pressure on the effective energy change $∆E^{\mathrm{eff}}=E_{\mathrm{sep}}^{\mathrm{eff}}-E_{\dim}^{\mathrm{eff}}$ upon dissociation of the coiled coil helices, namely, ${∆}_{p}∆E^{\mathrm{eff}}\equiv ∆E^{\mathrm{eff}}\left(8,000\;\mathrm{bar}\right)-∆E^{\mathrm{eff}}\left(1\;\mathrm{bar}\right)$, at 298 K, and its decomposition into nonpolar and polar parts. The numbers shown above the zero line are the values of ${∆}_{p}∆E^{\mathrm{eff}}$ (= ${∆}_{p}∆\mu^{\mathrm{ex}}$) and those of the nonpolar and polar parts

The effect of pressure on $∆E^{\mathrm{eff}}=E_{\mathrm{sep}}^{\mathrm{eff}}-E_{\dim}^{\mathrm{eff}}$ was examined; namely, ${∆}_{p}∆E^{\mathrm{eff}}\equiv ∆E^{\mathrm{eff}}\left(8000\;\mathrm{bar}\right)-∆E^{\mathrm{eff}}\left(1\;\mathrm{bar}\right)$, to verify the reliability of the present analysis. It is noteworthy that ${∆}_{p}∆E^{\mathrm{eff}}$ was equivalent to ${∆}_{p}∆\mu^{\mathrm{ex}}$ because $∆E^{\text{intra}}$ was independent of pressure. A few experiments on the pressure effect on GCN4‐p1 or related coiled coils are available^36^, ^45^, ^46^ for comparison with the theoretically obtained results. When GCN4‐p1 is denatured by heating, it has been observed by circular dichroism (CD) spectroscopy that two‐stranded coiled coil helices dissociate and become random coils.^36^ In contrast, infrared spectroscopy measurement has demonstrated that an increase in pressure stabilizes the helices of GCN4‐p1,^36^ as well as alanine‐based peptide (AK20).^47^ In addition, it has been observed by fluorescence spectroscopy that, by applying pressure, GCN4‐p1 coiled coil helices, as well as tropomyosin, dissociate so that they become two isolated helices.^45^ Taken together, these observations indicate that the two‐stranded coiled coil helices in GCN4‐p1 dissociated into two helical monomers with increasing pressure, and these helical monomers were stable at high pressure. A pressure‐induced stabilization of the separated state as ${∆}_{p}∆E^{\mathrm{eff}}=-28k_{B}T$ compared to the dimer state was observed (Figure 5a). The nonpolar part contribution to ${∆}_{p}∆\mu^{\mathrm{ex}}$ obtained by eliminating all GCN4‐p1 electric charges, ${∆}_{p}∆\mu_{\text{nonpol}}^{\mathrm{ex}}$, was the dominant part of ${∆}_{p}∆\mu^{\mathrm{ex}}$ (Figure 5a), indicating that electrostatic interactions did not affect the relative stabilization of the separated state at high pressure. The volume change due to the dissociation $∆V^{\mathrm{ex}}$ was obtained as −87 cm^3^/mol using a linear approximation for $V^{\mathrm{ex}}={\left(\partial \mu^{\mathrm{ex}}/\partial P\right)}_{T}$ (Table S3), which quantitatively agrees with the experimental value, −87 cm^3^/mol, determined by Silva and Cortines.^46^ These results, therefore, demonstrate that this theoretical calculation quantitatively reproduces the experimentally observed pressure‐induced separation of the coiled coil helices.^36^, ^45^ It would be worthy note that the geometrical void volume (*V*_v_) of the separated state is 128 cm^3^/mol smaller than that of the dimer state (Figure S2), indicating that the voids between the helices decrease by dissociation.

### 3.4 Concluding remarks

Recently, the theoretical solvation‐free energy analysis was performed for the model protein chignolin using an all‐atom molecular model.^26^ It was demonstrated that the folded state was dominantly stabilized by intramolecular interactions and that the water‐mediated interactions were rather unfavorable for folding. The native structure was determined by the balance between the opposing factors, namely the intramolecular direct interactions and the water‐mediated interactions, and the folded conformations were the result of the former slightly overcoming the latter. The derived intramolecular direct interaction mechanism contradicts the classical Kauzmann hydrophobic interaction hypothesis and the revised views by the OOMP method that acknowledged the dehydration of nonpolar groups as a minor stabilizing factor of the folded conformation.^2^, ^3^, ^17^, ^18^, ^19^, ^20^, ^21^ However, the model protein chignolin was small and did not have a hydrophobic interior core; thus, it was unsuitable for generalizing these conclusions to larger globule proteins.

In the present study, to investigate the energetics of the association of nonpolar groups buried in a protein interior, an effective energy analysis has been performed^30^ for leucine zipper interactions between two‐stranded GCN4‐p1 coiled coil helices. It was demonstrated that the direct interaction mechanism also held for the GCN4‐p1 leucine zipper interaction. The dispersion forces between the coiled coil helices overcame the unfavorable water‐mediated interactions; thereby, the coiled coil helical dimer was more stable than the separated helical monomers. Furthermore, the intramolecular direct interaction mechanism worked as the dominant factor in the folding of GCN4‐p1 from the unfolded coils. Taking all these results and arguments together, it was concluded that the energetics of protein folding relevant to nonpolar part contributions should be revised from the current view that the dehydration of nonpolar groups stabilizes proteins: (1) the dominant factor in hydrophobic core formation buried in the protein interior is not attributed to the water‐mediated interactions among the nonpolar groups, but to dispersion forces (van der Waals interactions between the nonpolar groups); and (2) the intramolecular direct and water‐mediated interactions, respectively, stabilized folded and unfolded conformations of protein. As a result of the competition between these opposing factors, the former slightly overcame the latter, so that the folded conformations were more stable than the unfolded ones. The dominant contribution of the van der Waals interactions between nonpolar groups to protein stabilities has already been drawn by the analyses using the OOMP method.^2^, ^3^, ^17^, ^18^, ^19^, ^20^, ^21^ However, the unfavorable water‐meditated interaction between nonpolar groups is distinct from their insights.^2^, ^3^, ^17^, ^18^, ^19^, ^20^, ^21^ The three‐dimensional‐reference interaction site model (3D‐RISM) analysis^48^ supports that water‐mediated interactions (total) are unfavorable for folding, although decomposition of the nonpolar part contributions was not conducted.

Why do dispersion forces between the helices overcome unfavorable water‐mediated interactions so that the dispersion forces work as the predominant factor in self‐association? Our method made a hypothetical nonpolar protein molecule by eliminating the charges on the protein (Figure 1c). For comparison, we focus on comparable large nonpolar molecules existing as carbon nanoparticles, such as fullerenes, carbon nanotubes, and graphene/graphite. It is phenomenologically well known that these nonpolar molecules are hardly dissolved in water. While it has often been supposed that water‐mediated interactions cause aggregation of these carbon materials, MD simulations have shown that water‐mediated interactions between carbon nanoparticles are commonly repulsive, and dispersion forces between them strongly aggregate them.^39^, ^40^ These computational results are consistent with the leucine zipper interaction for GCN4‐p1.

To address this issue, graphite was employed as a model carbon material that forms colloidal nanoparticles, and a quantity characterizing the strength of dispersion forces was investigated among the graphite nanoparticles and water molecules. The strength of the dispersion force between atoms in carbon materials may be quantified using the Lennard‐Jones (LJ) parameter $4\varepsilon \sigma^{6}$. The value of $4\varepsilon \sigma^{6}$ for the aromatic carbon atom in graphite^49^, ^50^ is nearly equivalent to that for liquid water^51^ (Table 1). However, the molar volume, $V_{m}$, of water is larger than $V_{m}$ of the carbon atom in graphite because the distance between oxygen atoms in liquid water (0.28 nm)^52^ is two times larger than that between aromatic carbon atoms for a graphene sheet (0.142 nm).^53^ As a result, the electron density of graphite is two times higher than that of bulk liquid water (Table 1). A dispersion force energy density parameter defined as $4\varepsilon \sigma^{6}/V_{m}$ was introduced, and the value for graphite (105.9 kcal·m^3^) is more than three times larger than that for liquid water (33.1 kcal·m^3^). Furthermore, if the geometric mean for these values is employed, which corresponds to the standard combining rule in the all‐atom optimized potentials for the liquid simulations (OPLS‐AA) force field,^50^ the dispersion force energy density for graphite‐water interaction is 59.2 kcal·m^3^, indicating that the parameter between graphite particles is ~2 times higher than that between the graphite particle and water. Consequently, the dispersion force between the graphite nanoparticles and water is unfavorable for aggregation and also makes the total water‐mediated interactions unfavorable. The dispersion force between the graphite nanoparticles stabilizes the aggregates because it overcomes the weaker dispersion force between the graphite nanoparticles and water.

**TABLE 1:** Dispersion force parameter of Lennard‐Jones (LJ) potential, $4\varepsilon \sigma^{6}$, molar volume, $V_{m}$, electron density, and a dispersion force energy density, $4\varepsilon \sigma^{6}/V_{m}$, for liquid water and graphite. The LJ parameter $4\varepsilon \sigma^{6}$ for water is taken from the TIP3P water model,^51^ and that for graphite is taken from the OPLS‐AA force field,^50^ according to a model of graphene and graphite.^49^ Based on the standard combining rule in the OPLS‐AA force field, $4\varepsilon \sigma^{6}/V_{m}$ between water and graphite is 59.2 kcal·m^3^, using the geometric mean of these parameters

	Liquid water	Graphite
LJ dispersion force parameter $4\varepsilon \sigma^{6}$ ([kcal/mol]·Å^6^)	595 ^51^	560 ^49^, ^50^
Molar volume $V_{m}$ (cm^3^/mol)	18	5.293 ^62^ for C atom
Electron density (nm^−3^)	335	683
Dispersion force energy density $4\varepsilon \sigma^{6}/V_{m}$ (kcal·m^3^)	33.1	105.9

Abbreviations: TIP3P, transferable intermolecular potential 3P; OPLS‐AA, all‐atom optimized potentials for liquid simulations.

Similar to graphite nanoparticles, the electron density of proteins is higher than that of bulk liquid water. In fact, a small‐angle X‐ray scattering of proteins in aqueous solution was observed. On the basis of these arguments, because of the higher electron density of nonpolar groups inside the protein interior as compared to bulk water, the dispersion forces between the nonpolar groups overcome the unfavorable water‐mediated interactions between them, resulting in the association of the nonpolar groups inside the protein in the native state. Taken together, it is reasonable to describe the underlying physical mechanism of the association of nonpolar groups in terms of the dispersion force.

The water‐mediated interactions between nonpolar particles (e.g., graphite) or between nonpolar groups in proteins are less significant for their association than we previously expected, and rather a destabilizer. This may be surprising in the field of protein science but is not in colloid science. In fact, colloid science has long successfully introduced the Derjaguin–Landau–Verwey–Overbeek (DLVO) theory to estimate colloidal stability, including protein aggregation.^54^, ^55^, ^56^ The DLVO theory describes the interaction potentials between colloids based on the balance between van der Waals attraction and electrostatic repulsion,^57^, ^58^ where aqueous solvents are modeled as a continuum dielectric medium that affects only electrostatic interactions. Hydration effects are regarded as correction factors termed non‐DLVO effects^59^, ^60^, ^61^; for example, hydrogen bonding with water and water‐mediated hydrophobic interactions is not taken into consideration.

In summary, the solvation‐free energy change upon leucine zipper formation in a coiled‐coil protein GCN4‐p1, a typical model for hydrophobic interactions, was examined. The accurate solvation‐free energy calculation using the RMDFT demonstrated that water‐mediated interactions were unfavorable for both the association of nonpolar groups and folding, while the dispersion forces between them were, instead, responsible for association and folding. This conclusion challenges the current view that the energetic cost of the hydration of nonpolar groups of proteins act as a stabilizing factor for folding. The robustness of the conclusion will be accepted through orthogonal theoretical and experimental analyses for other proteins in future works.

## 4 MATERIALS AND METHODS

### 4.1 MD simulations

MD simulations in the canonical (NVT) and isothermal–isobaric (NPT) ensembles were performed using the Gromacs 5.1.2 suite.^63^ The Amber99SB force field^64^ for GCN4‐p1 and the transferable intermolecular potential 3P (TIP3P) model^51^ for water were employed. The GCN4‐p1 leucine zipper initial configuration for starting the MD simulation was obtained from its X‐ray structure (Protein Data Bank identification code 2ZTA).^32^ The amino acid residue charge states were set to pH 7, employing charge neutral states for histidine and asparagine. A total of 18,050 and 16,775 water molecules were added to the cubic box under periodic boundary conditions for the MD simulations of the two‐stranded coiled coil helices and the monomer coil, respectively. The time step in the MD simulations was 2.0 fs. The temperature of 300 K and pressure of 1 bar were controlled by a Nosé–Hoover thermostat^65^, ^66^ and the Parrinello–Rahman barostat,^67^ respectively. Intramolecular bonds, including hydrogen atoms on GCN4‐p1, were constrained using the linear constraint solver for molecular simulations algorithm.^68^

### 4.2 RMDFT calculations

The solvation‐free energy of GCN4‐p1 for 100,000 helical dimer conformations, 100,000 isolated helical monomer conformations, and 100,000 monomeric coil conformations were calculated using the RMDFT accelerated by a graphics processing unit (GPU).^27^, ^28^, ^30^, ^69^ The standard error of all computational physical quantities was evaluated by decomposing the 100,000 conformational averages into five equal blocks. To calculate the site‐density distribution functions for water, we applied the three‐dimensional reference‐interaction‐site‐model (3D‐RISM) integral equation theory.^70^, ^71^ We employed the partially linearized HNC (PLHNC) equation,^70^ called the Kovalenko–Hirata (KH) equation,^71^ as the closure relations for both the 1D‐RISM equation for bulk water and the 3D‐RISM equation for solute–solvent systems. As for the model water in these RISM calculations, we used the TIP3P model with an additional LJ parameter for the hydrogen sites (*d*_H_ = 0.4 Å and $\varepsilon_{H}=0.046\;\text{kcal}/\mathrm{mol}$).^51^, ^72^ The solute–solvent cross parameters were deduced from the Lorentz–Berthelot mixing rule, $d_{\mathrm{ij}}=\left(d_{\mathrm{ii}}+d_{\mathrm{jj}}\right)/2$ and $\varepsilon_{\mathrm{ij}}=\sqrt{\varepsilon_{\mathrm{ii}}\varepsilon_{\mathrm{jj}}}$, commonly introduced as the solute–solvent combination rule in the RISM calculations. The 3D‐RISM integral equations were solved in a cubic cell with a size of 100 Å^3^ using a grid of 256^3^ points by utilizing graphics processing unit (GPU).^69^ In the same manner as our previous work,^30^ we used 0.00125 Å and 32,768 as the grid spacing and the number of grids, respectively, to solve the 1D‐RISM equation for bulk water and the effective‐density approximation (EDA) equation for the reference HS system.^73^ The number densities of water and the optimal HS diameters used for the thermodynamic states at 298 K, 1 bar and at 298 K, 8000 bar have been provided by our previous work.^30^

## CONFLICT OF INTEREST

The authors declare no competing interests.

## AUTHOR CONTRIBUTIONS

**Tomonari Sumi:** Conceptualization (lead); formal analysis (lead); funding acquisition (lead); investigation (equal); methodology (lead); project administration (lead); resources (lead); software (lead); supervision (lead); validation (lead); visualization (equal); writing—original draft (lead); writing—review and editing (equal). **Hiroshi Imamura:** Investigation (equal); validation (equal); visualization (equal); writing—review and editing (equal).

## Supporting information

**Appendix S1**: Supporting Information
