# Supplementary material for: Water‐mediated interactions destabilize proteins
Source: Protein Sci. 2021 Aug 20;30(10):2132–43. doi: 10.1002/pro.4168 (PMC8442971; doi:10.1002/pro.4168)
Supplement: Supplementary file 1 — Appendix S1: Supporting Information [file PRO-30-2132-s001.docx]

**Supporting Information for**

**Water-Mediated Interactions Destabilize Proteins**

**Tomonari Sumi^1,2^ and Hiroshi Imamura^3^**

^1^Research Institute for Interdisciplinary Science, Okayama University, 3-1-1 Tsushima-Naka, Kita-ku, Okayama 700-8530, Japan.

^2^Department of Chemistry, Faculty of Science, Okayama University, 3-1-1 Tsushima-Naka, Kita-ku, Okayama 700-8530, Japan.

^3^Department of Applied Chemistry, College of Life Sciences, Ritsumeikan University, Shiga 525-8577, Japan

**Figure S1. Effects of the charge elimination on the solvation-free energy (SFE) of the amino acid side-chain analogues in water.** SFE and SFE without charges are the almost identical for the non-polar aliphatic groups, while those show opposite signs for the aromatic and the polar groups. The following analog solutes were used: Leu, isobutane; Ile, *n*-butane; Val, propane; Ala, methane; Phe, toluene; Met, methyl ethyl sulfide; Cys, methanethiol; Trp, 3-methylindole; Thr, ethanol; Ser, methanol; Tyr, *p*-cresol; Gln, propionamide; Asn, acetamide; His(Hid), 4-Methylimidazole; His(Hie) 4-Methylimidazole. His(Hid) and His(Hie) indicate His with hydrogen on the delta nitrogen and His with hydrogen on the epsilon nitrogen, respectively.

**Figure S2. Void volume (*V*_v_) of the coil, dimeric and separated states of GCN4-p1.** *V*_v_ (per one molecule) was calculated using the program “ProteinVolume” developed by Chen and Makhatadze ^1^.

**Table S1.** **Change in the effective energy upon the dissociation of the helix dimer,** $\boldsymbol{\Delta}\boldsymbol{E}^{\text{eff}}\boldsymbol{=}\boldsymbol{E}_{\text{sep}}^{\text{eff}}\boldsymbol{-}\boldsymbol{E}_{\text{dim}}^{\boldsymbol{e}\text{ff}}$**, the intramolecular part** $\boldsymbol{\Delta}\boldsymbol{E}^{\text{intra}}$**, and the water-mediated part** $\boldsymbol{\Delta}\boldsymbol{\mu}^{\text{ex}}$**, at 298 K, 1 bar.** For comparison, the nonpolar part of these quantities is shown together. The values of $\mu^{\text{ex}}$ for the dimer and separated state are also listed. Standard error (SE) for these quantities is provided. The nonpolar part of $\mu^{\text{ex}}$ for the dimer and separated state is positive likewise hydrocarbons, whilst $\Delta\mu^{\text{ex}}$ is negative due to the dispersion forces between protein and water because the cavity formation energy which has a large positive value is almost canceled for the dimer and separated state.

| $k_{\text{B}}T$ | $\Delta E^{\text{intra}}$ | SE | $\Delta\mu^{\text{ex}}$ | SE | $\Delta E^{\text{eff}}$ | SE |
| --- | --- | --- | --- | --- | --- | --- |
| Total | 430 | 14 | –362 | 13 | 68 | 19 |
| Nonpolar part | 150 | 3 | –76 | 2 | 74 | 4 |
| kcal/mol | $\mu^{\text{ex}}$(dimer) | SE | $2\mu^{\text{ex}}$(helix) | SE |  |  |
| Total | –1220 | 2.5 | –1435 | 7.0 |  |  |
| Nonpolar part | 239 | 0.6 | 194 | 0.9 |  |  |

The values of Δ*E*^intra^, Δ *µ*^ex^, and Δ*E*^eff^ are listed in unit of *k*_B_*T*. The values of *µ*^ex^ for the dimer and separated state are listed in kcal/mol.

**Table S2.** **Change in the effective energy upon unfolding of the helix dimer into two coils** $\boldsymbol{\Delta}\boldsymbol{E}^{\text{eff}}\boldsymbol{=}\boldsymbol{E}_{\text{unfold}}^{\text{eff}}\boldsymbol{-}\boldsymbol{E}_{\text{fold}}^{\text{eff}}$**, the intramolecular part** $\boldsymbol{\Delta}\boldsymbol{E}^{\text{intra}}$**, and the solvent-mediated part** $\boldsymbol{\Delta}\boldsymbol{\mu}^{\text{ex}}$**, at 298 K, 1 bar.** The value of $\mu^{\text{ex}}$ for the monomer coil multiplied by 2 is also listed.

| $k_{\text{B}}T$ | $\Delta E^{\text{intra}}$ | SE | $\Delta\mu^{\text{ex}}$ | SE | $\Delta E^{\text{eff}}$ | SE |
| --- | --- | --- | --- | --- | --- | --- |
| Total | 127 | 38 | –61 | 30 | 66 | 49 |
| Nonpolar part | 114 | 6 | –62 | 3 | 52 | 7 |
| kcal/mol | ${2\mu}^{\text{ex}}$(coil) | SE |  |  |  |  |
| Total | –1256 | 17.8 |  |  |  |  |
| Nonpolar part | 202 | 1.6 |  |  |  |  |

The values of Δ*E*^intra^, Δ *µ*^ex^, and Δ*E*^eff^ are listed in unit of *k*_B_*T*. The values of *µ*^ex^ for the coil are listed in kcal/mol.

**Table S3.** **Excess partial molar volume of GCN4-p1 for the dimer and separated state.** These values are determined using a linear approximation between 1 bar and 8000 bar for $V^{\text{ex}}=\left( {\partial\mu^{\text{ex}}}/{\partial P} \right)_{T}$. The difference in $V^{\text{ex}}$ is defined as $\Delta V^{\text{ex}}=\left\langle V^{\text{ex}} \right\rangle_{\text{sep}}-\left\langle V^{\text{ex}} \right\rangle_{\text{dimer}}$. The nonpolar part of $V^{\text{ex}}$ is calculated from $V_{\text{nonpol}}^{\text{ex}}=\left( {\partial\mu_{\text{nonpol}}^{\text{ex}}}/{\partial P} \right)_{T}$.

| cm^3^/mol | Total | SE | Nonpolar part | SE |
| --- | --- | --- | --- | --- |
| dimer | 5755 | 19 | 6133 | 4 |
| separated | 5668 | 53 | 6047 | 6 |
| $\Delta V^{\text{ex}}$ | –87 | 56 | –86 | 7 |

**Appendix**

**The drawback of OOMP method**

In the Ooi–Oobatake ^2^ and Makhatadze–Privalov ^3,4^ method (OOMP method), the Gibbs energy of hydration of a protein ($\mu_{c}^{\text{ex}}$) with a conformation *c* is assumed to be proportional to the solvent-accessible surface area (ASA) of each functional group *k* of the protein:

$\mu_{c}^{\text{ex}}=\sum_{k}g_{k}{ASA}_{k}^{c}$ (A1)

where ${ASA}_{k}^{c}$ is the solvent-accessible surface area of group *k* at the conformation *c* of the protein and $g_{k}$is defined by

$g_{k}={\Delta G_{k}^{\text{hyd}}}/{{ASA}_{k}}$. (A2)

Here, $\Delta G_{k}^{\text{hyd}}$ is the Gibbs energy of hydration of the isolated group *k* and ${ASA}_{k}$ is its total solvent-accessible surface area. $\Delta G_{k}^{\text{hyd}}$ was estimated according to the data of the model compounds ^1-3^. *c* is the folded conformation (F) or the unfolded one (U). In this method, the buried functional groups inside the protein whose *ASA_k_* is zero do not contribute to the total Gibbs energy of hydration of the protein so that the cavity formation energy for the buried groups which yields a positive value are ignored. Such bias should manifest as unreasonable stabilization of the folded conformation with the lesser ASA of the buried groups as follows. Based on Eqs. A1 and A2, the Gibbs energy of hydration for protein unfolding (water-mediated interaction), $\Delta\mu^{\text{ex}}$, is written as

$\Delta\mu^{\text{ex}}\equiv\mu_{\text{U}}^{\text{ex}}-\mu_{\text{F}}^{\text{ex}}=\sum_{k}g_{k}({ASA}_{k}^{\text{U}}-{ASA}_{k}^{\text{F}})$, (A3)

where $\mu_{\text{U}}^{\text{ex}}$ and $\mu_{\text{F}}^{\text{ex}}$ are the Gibbs energy of hydration of unfolded and folded protein, and ${ASA}_{k}^{\text{U}}$ and ${ASA}_{k}^{\text{F}}$ are the solvent-accessible surface area of group *k* for the unfolded and folded protein. If the protein contains a functional group *k* buried inside, $\mu_{\text{F}}^{\text{ex}}$ does not involve the cavity formation energy of the buried group *k* because of the absence of the contribution from $g_{k}{ASA}_{k}^{\text{F}}$, while $\mu_{\text{U}}^{\text{ex}}$ involves that of the group *k* exposing to water through $g_{k}{ASA}_{k}^{\text{U}}$. Thus, the OOMP method always underestimate $\mu_{\text{F}}^{\text{ex}}$ compared with $\mu_{\text{U}}^{\text{ex}}$ due to the lack of the cavity formation energy for the functional groups buried inside the folded protein. The exposure of buried nonpolar groups to water with a positive value of $\Delta G_{k}^{\text{hyd}}$ is always regarded as unfavorable by the OOMP method. This should be the drawback of the ASA-dependent estimation of the Gibbs energy of protein hydration.

**References**

1. Chen CR, Makhatadze GI (2017) Molecular determinant of the effects of hydrostatic pressure on protein folding stability. Nature Communications 8:14561–9.

2. Ooi T, Oobatake M, Nemethy G, Scheraga HA (1987) Accessible Surface-Areas as a Measure of the Thermodynamic Parameters of Hydration of Peptides. Proc. Natl. Acad. Sci. U.S.A. 84:3086–3090.

3. Makhatadze GI, Privalov PL (1993) Contribution of hydration to protein folding thermodynamics. I. The enthalpy of hydration. J. Mol. Biol. 232:639–659.

4. Privalov PL, Makhatadze GI (1993) Contribution of hydration to protein folding thermodynamics. II. The entropy and Gibbs energy of hydration. J. Mol. Biol. 232:660–679.
